# *Zingiber officinale* Roscoe Rhizomes Attenuate Oxaliplatin-Induced Neuropathic Pain in Mice

**DOI:** 10.3390/molecules26030548

**Published:** 2021-01-21

**Authors:** Ji Hwan Lee, Daeun Min, Donghun Lee, Woojin Kim

**Affiliations:** 1 Department of Physiology, College of Korean Medicine, Kyung Hee University, Seoul 02453, Korea; mibdna@khu.ac.kr (J.H.L.); wndqhr1456@gmail.com (D.M.); 2 Department of Science in Korean Medicine, Graduate School, Kyung Hee University, Seoul 02453, Korea; 3 Department of Herbal Pharmacology, College of Korean Medicine, Gachon University, Gyeonggi-do 13120, Korea; dlee@gachon.ac.kr

**Keywords:** allodynia, chemotherapy-induced neuropathic pain, oxaliplatin, serotonin, *Zingiber officinale*

## Abstract

Oxaliplatin is a platinum derivative chemotherapeutic drug widely used against cancers, but even a single treatment can induce a severe allodynia that requires treatment interruption and dose diminution. The rhizome of *Zingiber officinale* roscoe (*Z. officinale*, ginger), has been widely used in traditional medicine to treat various diseases causing pain; however, its effect against oxaliplatin-induced neuropathic pain has never been assessed. In mice, a single oxaliplatin (6 mg/kg, i.p.) treatment induced significant cold and mechanical allodynia. Cold and mechanical allodynia were assessed by acetone drop and von Frey filament tests, respectively. Water extracts of *Z. officinale* (100, 300, and 500 mg/kg, p.o.) significantly attenuated both cold and mechanical allodynia induced by oxaliplatin. Intrathecal pre-treatment with the antagonist 5-HT_1A_ (NAN-190, i.t., 1 μg), but not with the antagonist 5-HT_2A_ (ketanserin, i.t., 1 μg), significantly blocked the analgesic effect of *Z. officinale* against both cold and mechanical allodynia. However, 5-HT_3_ antagonist (MDL-72222, i.t., 15 μg) administration only blocked the anti-allodynic effect of *Z. officinale* against cold allodynia. Real-time PCR analysis demonstrated that *Z. officinale* significantly increased the mRNA expression of the spinal 5-HT_1A_ receptor that was downregulated after oxaliplatin injection. These results suggest that *Z. officinale* may be a viable treatment option for oxaliplatin-induced neuropathic pain.

## 1. Introduction

Oxaliplatin is a widely used chemotherapeutic agent and was the first diaminocyclohexane platinum-based anti-cancer agent to be approved for the treatment of colorectal cancer [1]. Moreover, it has a better safety profile than cisplatin or carboplatin, which are other platinum-based drugs, as it bears no hepatoxicity or nephrotoxicity [2]. However, the use of oxaliplatin in cancer patients is limited due to the severe acute neuropathic pain induced even after a single injection in up to 80% of treated patients [3]. These paresthesia and dysesthesia can initiate acutely within 48 h after its administration [4] and are known to peak three days after its administration [5]. For the past several years, our lab has focused its efforts on understanding the mechanism of action of oxaliplatin-induced neuropathic pain [6,7] and on finding an effective treatment that bears no side effects [8,9,10]. However, an optimal drug has yet to be found, and the development of alternative strategies is continuously needed.

*Zingiber officinale* roscoe (*Z. officinale*) is a member of the Zingiberaceae family and is commonly known as ginger. For thousands of years, *Z. officinale* has been used worldwide, especially in East Asia to attenuate cold, headache, and digestive problems [11,12]. Chemical analysis show that *Z. officinale* contains more than 400 different compounds and, as major biologically active compounds, it includes gingerols, shogaols, and paradols [13]. In addition, amino acids, raw fiber, ash, protein, phytosterols, vitamins, and minerals are also reported to be present [14]. Experimental studies conducted in rodents reported that *Z. officinale* has various therapeutic effects such as anti-inflammatory [15], hypoglycemic [16], and anti-emetic [17] effects. Furthermore, in some animal models of pain, *Z. officinale* significantly attenuated muscle pain [18] and diabetes- and injury-induced neuropathic pain [19,20], showing therapeutic potential in inhibiting chemotherapy-induced neuropathic pain. However, to date, its effect against oxaliplatin-induced neuropathic pain has never been assessed.

Serotonin (5-hydroxytryptamine; 5-HT) is a small-molecule neurotransmitter, which is known to be involved in the descending pain inhibitory system [21,22]. It is known to be synthesized in the rostro ventromedial medulla (RVM), and axons of serotonergic neurons are present in the descending pathway to the spinal cord. It is known that 5-HT is able to interact with 7 different classes of receptors that are differentiated into 15 subtypes [23]. Although most of these receptors are present in the spinal dorsal horn neurons, their involvement in pain modulation remains largely unknown, and studies conducted on pain have been mainly focused on 5-HT_1,_ 5-HT_2,_ and 5-HT_3_ receptors [24]. However, even the role of these receptors in pain attenuation remains to be elucidated, as their involvements appears inconsistent depending on the disease models and drugs used [25]. In oxaliplatin-induced neuropathic pain, spinal 5-HT receptors have also been reported to play an important role, as the analgesic actions of various treatments were blocked by pre-treatment with spinal 5-HT receptor antagonists [8,26,27]. Furthermore, the American Society of Clinical Oncology has recommended duloxetine, a serotonin and norepinephrine reuptake inhibitor, to be used against oxaliplatin-induced neuropathic pain [28]. However, although duloxetine is known to possess more selective re-uptake inhibition and binding of the 5-HT transporter with respect to the norepinephrine transporter [29], the role of 5-HTergic neurotransmission in oxaliplatin-induced neuropathic pain is still a controversial issue [30], and further investigation is needed.

Thus, the aim of this study was, first, to assess the analgesic effect of *Z. officinale* on oxaliplatin-induced neuropathic pain. Second, to assess the involvement of spinal 5-HT receptors in the anti-allodynic effect of *Z. officinale*. Finally, to analyze whether the messenger RNA (mRNA) expression level of spinal 5-HT receptors could be modified following oxaliplatin and *Z. officinale* treatments.

## 2. Results

### 2.1. Oxaliplatin Administration Induces Cold and Mechanical Allodynia in Mice

**Figure 1:**
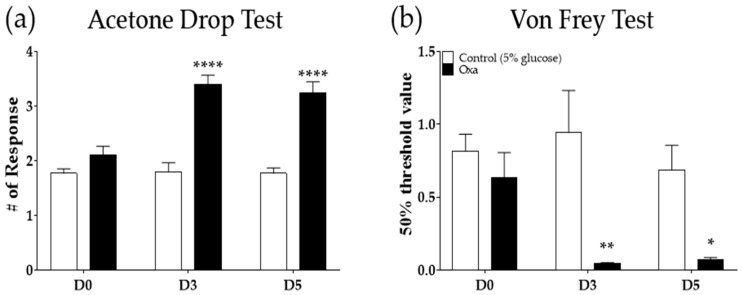
Induction of cold and mechanical allodynia by a single intraperitoneal injection of oxaliplatin in mice. Three to 5 days after a single injection of oxaliplatin (6 mg/kg, i.p.), cold (**a**) and mechanical (**b**) allodynia were produced. Cold and mechanical allodynia were assessed by using the acetone drop and von Frey filament tests, respectively. Control mice were treated with a 5% glucose solution (i.p.). Oxa: oxaliplatin, D0: before the injection of oxaliplatin or 5% glucose, D3: 3 days after the injection of oxaliplatin or 5% glucose, D5: 5 days following the administration of oxaliplatin or 5% glucose. Control (5% glucose): *n =* 5, oxaliplatin: *n =* 5. * *p* < 0.05, ** *p <* 0.01, **** *p <* 0.0001 vs. Control with two-way ANOVA followed by Sidak’s post-test for multiple comparisons.

Following a single intraperitoneal injection of oxaliplatin (6 mg/kg), significant cold and mechanical allodynia were induced on day three (D3) and day five (D5) (Figure 1a,b). These results are in accordance with our previous study showing that a single oxaliplatin treatment induced cold and mechanical allodynia 3 to 7 days after injection [8]. On D3 and D5, cold allodynia was strongly induced in oxaliplatin-injected mice compared to mice treated with 5% glucose (control, *p* < 0.0001), as shown in Figure 1a. In the von Frey test, the 50% threshold value also significantly lowered both on D3 (*p* < 0.01) and on D5 (*p <* 0.05) after oxaliplatin injection compared to control (Figure 1b). Cold allodynia was measured by using the acetone drop test, and mechanical allodynia was assessed by using the von Frey filament test.

### 2.2. Anti-Allodynic Effect of Z. officinale on Oxaliplatin-Induced Neuropathic Pain in Mice

**Figure 2:**
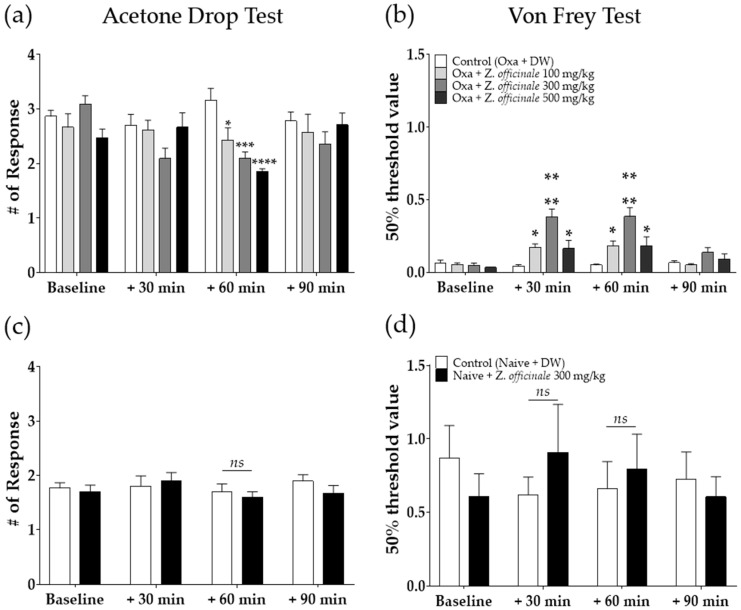
Time-dependent effect of *Zingiber officinale* on cold and mechanical allodynia induced by oxaliplatin (**a**,**b**). Time-dependent effect of *Z. officinale* in naïve mice (**c**,**d**). The effect of the oral administration of *Z. officinale* on cold (**a**) and mechanical (**b**) allodynia induced by a single oxaliplatin (6 mg/kg, i.p.) injection. Cold and mechanical allodynia were assessed by using the acetone drop and von Frey filament tests, respectively. Effect of *Z. officnale* administration (p.o.) on cold (**c**) and mechanical (**d**) stimuli in naïve mice. For cold and mechanical stimuli, acetone drop and von Frey filament tests were used, respectively. Distilled water (DW) was used as control. Control (Oxa + DW): *n* = 10, Oxa + *Z. officinale* 100 mg/kg: *n* = 7, Oxa + *Z. officinale* 300 mg/kg: *n* = 7, Oxa + *Z. officinale* 500 mg/kg: *n* = 7 (**a**,**b**). Control (Naïve + DW): *n* = 5, Naïve + *Z. officinale* 300 mg/kg: *n* = 5 (**c**,**d**). Baseline: before the injection of DW or *Z. officinale*, *ns*: non-significant, * *p* < 0.05, ** *p* < 0.01, *** *p* < 0.001, **** *p* < 0.0001 vs. Control with two-way ANOVA followed by Sidak’s post-test for multiple comparisons.

To observe the analgesic effect of *Z. officinale* on oxaliplatin-induced cold and mechanical allodynia, three different doses of *Z. officinale* (100, 300, or 500 mg/kg) were administered orally at D3, when significant cold and mechanical allodynia were evident in mice. Behavioral tests were conducted before and 30 min, 60 min, and 90 min after the injection of *Z. officinale*. The results showed significant alleviation of cold allodynia in a dose-dependent manner 60 min after *Z. officinale* treatment (Figure 2a). *Z. officinale* treatment also caused a significant increase of the 50% threshold value compared to control (distilled water; DW), which began 30 min after the injection and lasted for 60 min (Figure 2b). However, for both cold and mechanical allodynia, the analgesic effect of *Z. officinale* disappeared after 90 min. In our following experiments, 300 mg/kg of *Z. officinale* was used. Furthermore, the same dose of *Z. officinale* (300 mg/kg, p.o.) treatment in naïve mice induced no significant change in behavioral responses in the acetone drop and von Frey filament tests compared to DW-injected mice (Figure 2c,d).

### 2.3. Spinal 5-HT Receptors Are Involved in the Neuropathic Pain-Alleviating Effect of Z. officinale

**Figure 3:**
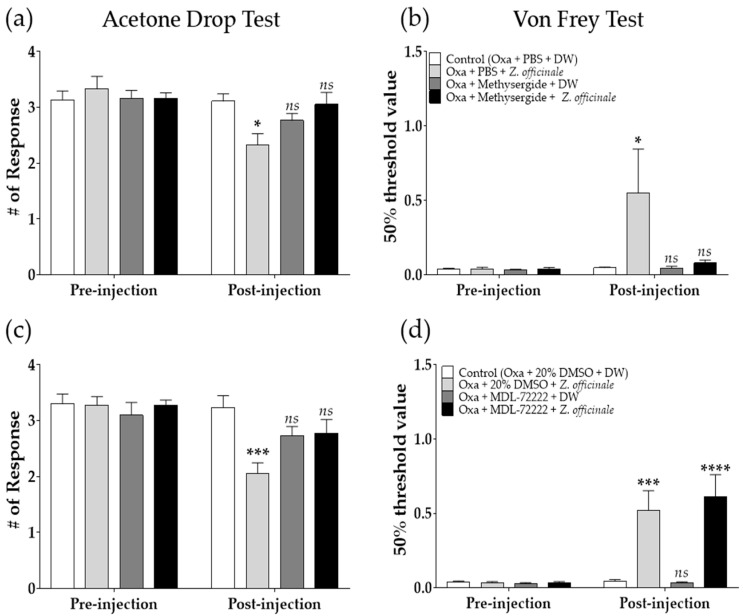
Effect of intrathecal administration of serotonin receptor antagonists on the analgesic effect of *Z. officinale* against oxaliplatin-induced neuropathic pain. The effect of methysergide (**a**,**b**) and MDL-72222 (**c**,**d**) on *Z. officinale*-induced inhibition of cold (**a,c**) and mechanical (**b**,**d**) allodynia. When significant allodynic signs were observed in oxaliplatin-treated mice, methysergide or MDL-72222 was injected intrathecally in mice. PBS or 20% DMSO was used as a control for methysergide and MDL-72222, respectively. *Z. officinale* or DW was administered orally 20 min after the administration of methysergide, MDL-72222, PBS, or 20% DMSO. Pre-injection: before the injection of PBS, methysergide, 20% DMSO, MDL-7222, and *Z. officinale*. Post-injection: After the injection of PBS, methysergide, 20% DMSO, MDL-7222, and *Z. officinale*. Cold and mechanical allodynia were assessed by using the acetone drop and von Frey filament tests, respectively. Control (Oxa + PBS + DW): *n* = 6, Oxa + PBS + *Z. officinale*: *n* = 6, Oxa + Methysergide + DW: *n* = 5, Oxa + Methysergide + *Z. officinale*: *n* = 6 (a, b). Control (Oxa + 20% DMSO + DW): *n* = 6, Oxa + 20% DMSO + *Z. officinale*: *n* = 6, Oxa + MDL-72222 + DW: *n* = 6, Oxa + MDL-72222 + *Z. officinale*: *n* = 6 (c, d). *ns*: non-significant, * *p* < 0.05, *** *p* < 0.001, **** *p* < 0.0001 vs. Control (Oxa + PBS + DW or Oxa + 20% DMSO + DW) with two-way ANOVA followed by Tukey’s post-test for multiple comparisons.

To determine whether spinal 5-HT receptors are involved in the effect of *Z. officinale* against oxaliplatin-induced neuropathic pain, methysergide (mixed 5-HT_1_ and 5-HT_2_ antagonists) or MDL-72222 (5-HT_3_ antagonist) was intrathecally injected 20 min before *Z. officinale* treatment, as explained in materials and methods. Our results showed that the analgesic effects of *Z. officinale* on oxaliplatin-induced cold and mechanical allodynia were significantly blocked by methysergide pre-treatment, as the effect of *Z. officinale* was nullified in the Oxa + methysergide + *Z. officinale* group (Figure 3a,b). However, MDL-72222 failed to completely block the analgesic action of *Z. officinale*, as only cold but not mechanical allodynia was nullified after *Z. officinale* treatment (Figure 3c,d). These results showed that spinal 5-HT_1_ and 5-HT_2_ receptors are involved in the anti-allodynic effect of *Z. officinale* against both cold and mechanical allodynia, whereas 5-HT_3_ receptors are only involved in its anti-allodynic effect against cold allodynia. As 5-HT_3_ receptors are only partially involved in the pain-alleviating effect of *Z. officinale*, the next study was focused on spinal 5-HT_1_ and 5-HT_2_ receptors.

### 2.4. Spinal 5-HT_1A_ but Not 5-HT_2A_ is Involved in the Anti-Allodynic Effect of Z. officinale

**Figure 4:**
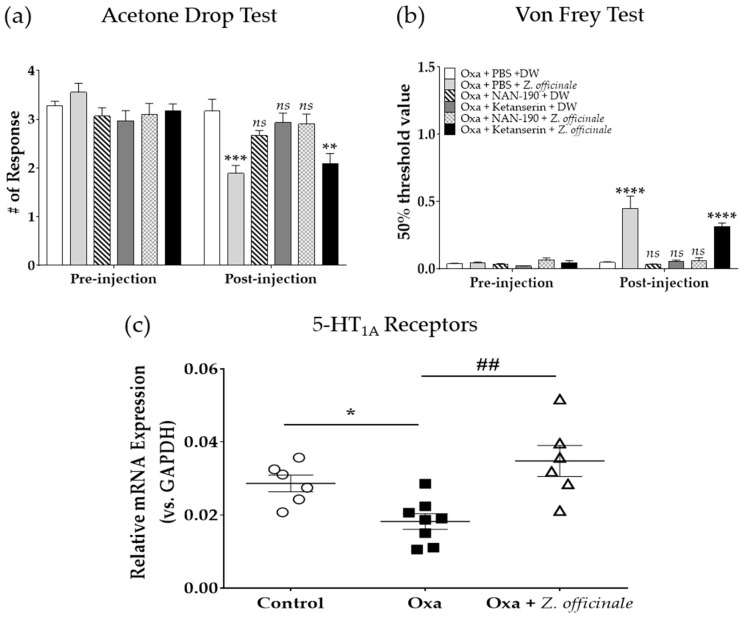
Effect of intrathecal injection of NAN-190 or ketanserin on *Z. officinale*-dependent inhibition of oxaliplatin-induced neuropathic pain in mice (**a**,**b**). Spinal mRNA expression level of 5-HT_1A_ receptors before and after oxaliplatin and *Z. officinale* treatment (**c**). Cold (**a**) and mechanical (**b**) allodynia were assessed by using the acetone drop and von Frey filament tests, respectively. On day 3 following oxaliplatin injection, NAN-190 or ketanserin was injected intrathecally in mice. PBS was used as a control for both NAN-190 and ketanserin. The relative mRNA expression of 5-HT_1A_ was measured before and after oxaliplatin injection and *Z. officinale* treatment (**c**). Control (Oxa + PBS + DW): *n* = 6, Oxa + PBS + *Z. officinale*: *n* = 6, Oxa + NAN-190 + DW: *n* = 5, Oxa + Ketanserin + DW: *n* = 5, Oxa + NAN-190 + *Z. officinale*: *n* = 7, Oxa + Ketanserin + *Z. officinale*: *n* = 7. *ns*: non-significant, ** *p* < 0.01, *** *p* < 0.001, **** *p* < 0.0001 vs. Control (Oxa + PBS + DW) with two-way ANOVA followed by Tukey’s post-test for multiple comparisons (**a**,**b**). Control: *n* = 6, Oxa: *n* = 8, Oxa + *Z. officinale*: *n* = 6. * *p* < 0.05 vs. Control, ## *p* < 0.01 vs. Oxa with one-way ANOVA followed by Tukey’s post-test for multiple comparisons (**c**).

As spinal 5-HT_1_ and 5-HT_2_ receptors were shown to be involved in the analgesic effect of *Z. officinale* against both cold and mechanical allodynia, the next experiments were conducted by using 5-HT_1A_ (NAN-190) or 5-HT_2A_ (ketanserin) antagonists. As reported in the literature, among various 5-HT_1_ and 5-HT_2_ receptors subtypes, both 5-HT_1A_ and 5-HT_2A_ are known to be deeply involved in pain modulation [24]. NAN-190 or ketanserin was injected 20 min prior to *Z. officinale* administration. Pre-treatment with NAN-190 strongly blocked the anti-allodynic effect of *Z. officinale* on both cold and mechanical allodynia, whereas ketanserin failed to block this effect (Figure 4a,b). This result showed that spinal 5-HT_1A_ but not 5-HT_2A_ receptors are involved in the analgesic effect of *Z. officinale*.

Subsequently, to assess whether the mRNA expression level of spinal 5-HT_1A_ receptor was altered after oxaliplatin and *Z. officinale* treatment, real-time PCR was conducted to quantify the mRNA expression level of 5-HT_1A_ receptor in the spinal cord. The results showed that the relative mRNA expression level (ratio of 5-HT_1A_ receptor mRNA to GAPDH mRNA) of spinal 5-HT_1A_ receptor was significantly decreased after oxaliplatin treatment (control vs. Oxa, *p* < 0.05), whereas *Z. officinale* administration significantly up-regulated the decreased mRNA expression level of 5-HT_1A_ receptor (Oxa vs. Oxa + *Z. officinale, p* < 0.01), as shown in Figure 4c. This indicated that the expression level of spinal 5-HT_1A_ receptor can be modulated by oral administration of *Z. officinale*.

### 2.5. Identification of Active Components in Z. officinale by Using HPLC

**Figure 5:**
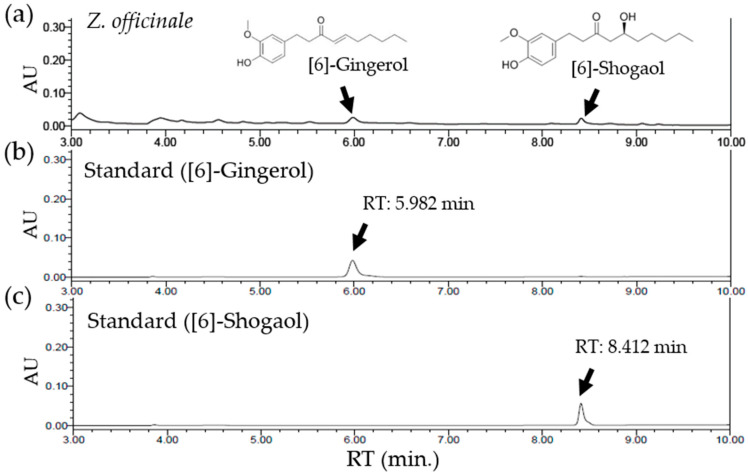
Quantification of chemicals in *Z. officinale* using HPLC. HPLC chromatograms of a water extract of *Z. officinale* (**a**) and of [6]-gingerol (**b**) and [6]-shogaol (**c**) as standards. Two peaks in (**a**) represent [6]-gingerol (5.982 min) and [6]-shogaol (8.412 min), sequentially. The standard ultraviolet detection wavelength was set at 230 nm. The *X*-axis reports the retention time (RT), and the *Y*-axis the absorbance unit (AU).

To quantify the components of *Z. officinale* contributing to the attenuation of oxaliplatin-induced neuropathic pain, HPLC was conducted. By using an established HPLC method, the presence of [6]-gingerol and [6]-shogaol in *Z. officinale* was confirmed. The retention times of [6]-gingerol and [6]-shogaol from *Z. officinale* were 5.982 min and 8.412 min, respectively. The regression equation of [6]-gingerol and [6]-shogaol and the estimated correlation coefficients (*r*^2^) of each phytochemical were determined based on the plots of peak value (*y*) versus concentrations (*x*). The regression equation of [6]-gingerol was *y* = 0.000138*x* − 0.00140 (*r*^2^ = 0.999765). For [6]-shogaol, it was *y* = 0.000107*x* + 0.00354 (*r*^2^ = 0.999600). The content of [6]-gingerol and [6]-shogaol in a water extract of *Z. officinale* was 0.012432% (310.8 μg/g) and 0.007251% (181.275 μg/g), respectively (Figure 5).

## 3. Discussion

In this study, we showed for the first time that oral administration of *Z. officinale* could significantly attenuate allodynia induced by a single oxaliplatin treatment. On cold allodynia, *Z. officinale* showed dose-dependent (100, 300, and 500 mg/kg) analgesic effects, whereas on mechanical allodynia, the dose of 300 mg/kg was shown to exhibit the strongest effect. In addition, we demonstrated that spinal 5-HT_1A_ but not 5-HT_2A_ receptors were involved in *Z. officinale*-induced analgesia against cold and mechanical allodynia. 5-HT_3_ receptors were only involved in the cold allodynia-alleviating effect of *Z. officinale*. Finally, we demonstrated that *Z. officinale* treatment could significantly increase the mRNA expression of 5-HT_1A_ receptors in the spinal cord, which was down-regulated by oxaliplatin treatment.

Although oxaliplatin is a widely used chemotherapeutic drug, severe peripheral neuropathy induced even after a single injection can decrease the quality of life of the treated patients and may endanger the life of the patients as it can interrupt the treatment schedule. Various mechanisms have been proposed, such as malfunction of voltage-gated sodium [6] and potassium channels [31] in the dorsal root ganglia (DRG) neuronal cells, a change in glial activities [7], and mitochondria dysfunction in peripheral neurons [32]. However, oxaliplatin mechanism of action is not clearly understood, and an alternative effective drug has yet to be developed.

*Z. officinale* has been widely used from the antiquity as a condiment and herbal medicine. Its therapeutic effects against various diseases have been reported, and some studies also focused on its analgesic effect. Sepehvand et al. showed that intraperitoneal treatment with *Z. officinale* is able to potentiate morphine-induced analgesia by enhancing the descending pain inhibitory system in rats [11]. Borgonetti et al. reported that oral administration of *Z. officinale* could decrease spinal nerve injury-induced neuropathic pain by inhibiting neuro-inflammation in mice [20]. In accordance with these studies, our results demonstrated that oral administration of *Z. officinale* could significantly attenuate oxaliplatin-induced cold and mechanical allodynia in mice. The main biologically active components of *Z. officinale* include [6]-gingerol and [6]-shogaol, which are both known to possess analgesic effects in various animal models of pain [33,34,35]. Furthermore, by using a parallel artificial membrane permeability assay for the blood brain barrier (BBB), both [6]-gingerol and [6]-shogaol were shown to be able to passively cross the BBB [36], suggesting that *Z. officinale* may directly act on the central nervous system (CNS). Moreover, the two compounds were shown to be extremely rapidly absorbed and eliminated following a single administration, as 2 h after their administration, their plasma levels were significantly decreased [37]. This may explain how a single *Z. officinale* administration could acutely decrease oxaliplatin-induced neuropathic pain and increase the mRNA expression of the 5-HT receptor in the spinal cord.

In our study, intrathecal pre-treatment with non-selective spinal 5-HT_1_ and 5-HT_2_ receptors antagonist (methysergide) significantly blocked the anti-allodynic effect of *Z. officinale* against both cold and mechanical allodynia, whereas the 5-HT_3_ receptor antagonist (MDL-72222) only blocked *Z. officinale* effect against cold allodynia. It has been reported that 5-HT_3_ receptors are mostly present in unmyelinated C-fibers of the spinal dorsal horn [38]. However, according to data obtained from others [6] and our lab [10], oxaliplatin-induced neuropathic pain is shown to be mostly mediated by myelinated A-fibers rather than by unmyelinated C-fibers [6,10]. Furthermore, in several studies, cold allodynia is reported to be mediated mostly by unmyelinated fibers [39]. This may explain why blocking 5-HT_3_ receptors only partially attenuated the analgesic effect of *Z. officinale*. Thus, in our following experiments, we focused on spinal 5-HT_1_ and 5-HT_2_ receptors, as they were shown to be involved in the alleviating effect of *Z. officinale* against both cold and mechanical allodynia.

Our results demonstrated that spinal 5-HT_1A_ but not 5-HT_2A_ receptors are involved in the analgesic effect of *Z. officinale*, as only the 5-HT_1A_ receptor antagonist (NAN-190) completely blocked the analgesic action of *Z. officinale* against both cold and mechanical allodynia induced by oxaliplatin. In addition, the quantification of 5-HT_1A_ receptors by real-time PCR demonstrated that *Z. officinale* significantly increased the lowered mRNA expression level of spinal 5-HT_1A_ receptor. The 5-HT_1A_ receptor is a G protein-coupled receptor widely present in the CNS. In the spinal cord, 5-HT_1A_ receptors are known to be expressed by spinothalamic interneurons in the superficial as well as deeper laminae and by gamma-aminobutyric acid(GABA)ergic interneurons in the spinal dorsal horn [40,41]. Various studies have reported that a spinal injection of the 5-HT_1A_ receptor agonist 8-hydroxy-2-(di-***n***-propylamino)tetralin resulted in attenuation of pain [42,43]. Morphological studies have reported that spinal 5-HT_2A_ receptors are also present in the laminae I–IV of the spinal dorsal horn; however, contrary to spinal 5-HT_1A_ receptors, 5-HT_2A_ receptors are reported to be involved in the development of inflammatory and neuropathic pain [44]. In a vincristine-induced neuropathic pain animal model, it was shown that 5-HT_2A_^−/−^ mutant mice did not develop vincristine-induced allodynia or hypersensitivity, whereas their 5-HT_2A_^+/+^ littermates showed significant signs of pain. Furthermore, epidural injection a 5-HT_2A_ receptor antagonist attenuated thermal and mechanical allodynia, showing that spinal 5-HT_2A_ receptors are more involved in facilitating than in inhibiting the effect of pain [45]. Altogether, these results indicate that spinal 5-HT_1A_ receptors may play an important role in the anti-allodynic effect of *Z. officinale*. In line with this, Nievergelt et al. reported that various sub-components of ginger can interact with the human 5-HT_1A_ receptor with significant to moderate binding affinities [46].

## 4. Materials and Methods

### 4.1. Animals

Adult C57BL/6 mice (6 weeks old) were obtained from Daehan biolink (Chungbuk, Korea) and housed in a specific pathogen-free animal center. Animals were randomly distributed in cages. They were kept in a room with a temperature of 23 ± 2 °C, humidity of 65 ± 5%, fixed 12 h light/dark cycle, and with food and water ad libitum. All experimental protocols were approved by the Kyung Hee University Animal Care and Use Committee (KHUASP(SE)-20-448) on 15 November 2020 and were conducted in accordance with the guidelines of the International Association for the Study of Pain [47].

### 4.2. Oxaliplatin Administration

Oxaliplatin (Sigma Aldrich, St. Louis, MO, USA) was dissolved in a 5% glucose solution at a concentration of 2 mg/mL, as in our previous study [10]. Oxaliplatin was administered to mice intraperitoneally in an amount of 6 mg/kg. For control, the same amount of a 5% glucose solution was used. To assess whether oxaliplatin administration induced cold and mechanical allodynia in mice, behavioral tests were conducted before (baseline), 3 (D3), and 5 (D5) days after its injection.

### 4.3. Preparation of and Treatment with Z. Officinale

The dried root of *Z. officinale* (*rhizome of Zingiber officinale roscoe*) used in the experiments was procured from Yaksudang Pharmaceutical limited company (Seoul, Korea). The voucher specimen number was deposited as D200909001. *Z. officinale* was extracted using a reflux apparatus (distilled water (DW), 3 h at 100 °C). The extracted solution was filtered and condensed using a low-pressure evaporator. The *Z. officinale* extract had a yield of 17.53% after freeze-drying at −80 °C. The lyophilized *Z. officinale* powder was diluted in DW to obtain a stock solution of 80 mg/mL. *Z. officinale* was orally administered at three different doses (100, 300, and 500 mg/kg). The same volume (0.3 mL) of DW was orally administered to control animals.

### 4.4. Treatment with Serotonergic Antagonists

To assess the involvement of spinal serotonergic receptors in the analgesic effect of *Z. Officinale* against oxaliplatin-induced neuropathic pain, serotonin receptor antagonists were intrathecally injected 20 min before the oral administration of *Z. officinale*. The 5-HT receptor antagonists used in the experiments were methysergide (mixed 5-HT_1_ and _2_ receptor antagonists, 10 µg, concentration 2 µg/µL), NAN-190 (5-HT_1A_ receptor antagonist, 1 µg, concentration 0.2 µg/µL), ketanserin (5-HT_2A_ receptor antagonist, 1 µg, concentration 0.2 µg/µL), and MDL-72222 (3-tropanyl-3,5-dichlorobenzoate, 5-HT_3_ receptor antagonist, 15 µg, concentration 3 µg/µl). All antagonists used in this study were purchased from Tocris (Cookson, UK). Under isoflurane anesthesia (2–2.5% in N_2_O/O_2_: 1:1 *v*/*v*), 5 μL of antagonist solutions was injected at the lumbar 5–6 intervertebral level by using a Hamilton syringe (Hamilton Company, Reno, NV, USA) to deliver the drug directly into the subarachnoid space, as in our previous study [26]. For control, 5 μL of solvents (PBS or 20% dimethyl sulfoxide (DMSO)) was injected.

### 4.5. Behavioral Assessments

Behavior tests were conducted to assess the degree of allodynia in mice, as in our previous study [10]. Cold allodynia and mechanical allodynia were measured by using the acetone drop and von Frey filament tests, respectively. For acclimation, the animals were placed on a metal mesh floor and were caged in an inverted clear plastic cage (12 × 8 × 6 cm^3^) for 30 min before all behavioral tests. To assess the behavioral responses against cold stimuli, an acetone drop (10 μL) was applied on the mid-plantar hind paw of the mice. The behavioral responses (flicking and licking) against the stimulus were observed and counted for 30 sec. Thus, the term “# of response” mentioned in the figures stands for the average number of responses to an acetone drop of 10 μL, counted for 30 s.

To measure mechanical allodynia, a series of von Frey filaments (bending force of 0.02, 0.04, 0.07, 0.16, 0.4, 0.6, 1, 1.4, 2 g, Stoelting, Kiel, WI, USA) were applied on the mid-plantar hind paws. The Dixon’s up-down method and Chaplan’s calculation method were used, and a withdrawal threshold of 15 g was applied as the cut-off [48,49]. The results obtained from both hind paws were averaged.

### 4.6. Behavioral Tests Schedules

Behavioral tests were conducted with different timelines in each experiment. To measure the analgesic effect of *Z. officinale* against oxaliplatin-induced cold and mechanical allodynia, behavioral tests were conducted before (baseline) and 30, 60, and 90 min after *Z. officinale* oral administration on day 3 following oxaliplatin injection. To determine the role of spinal 5-HT receptors in the analgesic effects of *Z. officinale*, behavioral tests were conducted before the injection of antagonists (pre-injection) and 60 min after *Z. officinale* treatment (post-injection). *Z. officinale* was administered 20 min after the intrathecal injection of the antagonists.

### 4.7. High-Performance Liquid Chromatography (HPLC) Analysis of the Z. officinale Extract

A quantitative analysis of *Z. officinale* was carried out by the HPLC alliance system, with a 2998 photodiode array detector (Waters, Milford, MA, USA). Separation was done at 30 °C on a C_18_ column (Waters, Milford, MA, USA; 5 μm, 150 × 4.6 mm^2^). The sample injection volume was 10 μL. The compounds [6]-gingerol (2 mg/mL) and [6]-shogaol (2 mg/mL) were dissolved in acetonenitrile and used as standards to qualify and quantify components of *Z. officinale*. At a flow rate of 1.1 mL/min, mobile phase A (water) and mobile phase B (acetonenitrile) were operated using a gradient program (0–1.5 min, hold 35% B; 1.5–1.8 min, from 35 to 60% B; 1.8–5 min, hold 60% B; 5–6.5 min, from 60 to 100% B; 6.5–9 min, hold 100% B; 9–9.1 min, 100–35% B; 9.1–12 min, hold 35% B). The standard ultraviolet detector wavelength was set at 230 nm [50].

### 4.8. RNA Extraction and Real-Time PCR

When *Z. officinale* induced significant attenuation of cold and mechanical allodynia, animals were perfused with PBS, and the lumbar segments of the spinal cord were collected. Collected spinal cords were homogenized with Easy Blue (Intron Company, Seongnam, Korea) solution. RNA was extracted from the spinal cord, following the manufacturer’s protocol. The extracted RNA was qualified and quantified by NanoDrop (ThermoFisher, Waltham, MA, USA) and converted to complementary DNA (cDNA) using a cDNA synthesis kit (Bioneer Corporation, Daejeon, Korea). The mRNA level of 5-HT_1A_ (*HTR1A*) was determined with SYBR Green qPCR Mastermix (Bioline Reagents Ltd., London, United Kingdom) in a CFX Connect Real-Time PCR system (Bio-Rad, Laboratories Inc., Hercules, CA, USA). Data are expressed as the ratio of targeted mRNA to glyceraldehyde 3-phosphate dehydrogenase (GAPDH) mRNA (relative mRNA expression). The following table lists the PCR primers used in the experiments (Table 1).

### 4.9. Statistical Analysis

All data were presented as mean ± standard error of the mean (SEM). Statistical analysis and graphic works were performed by using Prism 7.0 (GraphPad software, La Jolla, CA, USA). Two-way ANOVA (analysis of variance) followed by Sidak’s or Tukey’s post-test for multiple comparisons and student t-test were used for statistical analyses. In all cases, *p* < 0.05 was considered to indicate significant differences.

## 5. Conclusions

In conclusion, our study demonstrated that the oral administration of different doses of *Z. officinale* could significantly attenuate oxaliplatin-induced cold and mechanical allodynia and that both spinal 5-HT_1A_ and 5-HT_3_ receptors are involved in *Z. officinale* analgesic effect against cold allodynia, whereas only spinal 5-HT_1A_, but not 5-HT_3_ receptors, are involved against mechanical allodynia. In addition, *Z. officinale* administration could increase the mRNA expression level of spinal 5-HT_1A_ receptors that was reduced after oxaliplatin treatment. Future studies are needed to clearly elucidate the involvement of the 5-HT system in the analgesic action of *Z. officinale* and its sub-components against oxaliplatin-induced neuropathic pain.

## 6. Patent

The content of this article are related to a patent application in Korea (10-2020-0189163).

## Figures and Tables

**Table 1:** PCR primer sequences for PCR analysis.

Type	Sequence
GAPDH (Forward)	5′-GGAGGTAGCTCCTGATTCGC-3′
GAPDH (Reverse)	5′-CACATTGGGGGTAGGAACAC-3′
HTR1A (Forward)	5′-TACTCCACTTTCGGCGCTTT-3′
HTR1A (Reverse)	5′-GGAGGTAGCTCCTGATTCGC-3′

## Data Availability

The data presented in this study are openly available.

## Sample Availability

Sample of water extract of *Z. officinale* is available from the authors (voucher specimen number was deposited as D200909001).
